# Coxsackievirus A6 2C protein antagonizes IFN-β production through MDA5 and RIG-I depletion

**DOI:** 10.1128/jvi.01075-23

**Published:** 2023-10-17

**Authors:** Shao-Hua Wang, Juan Du, Jinghua Yu, Yifei Zhao, Yu Wang, Shucheng Hua, Ke Zhao

**Affiliations:** 1 Center of Infectious Diseases and Pathogen Biology, First Hospital of Jilin University, Changchun, China; 2 Institute of Virology and AIDS Research, First Hospital of Jilin University, Changchun, China; 3 Department of Respiratory Medicine, First Hospital of Jilin University, Changchun, China; St. Jude Children's Research Hospital, Memphis, Tennessee, USA

**Keywords:** coxsackievirus A6, enterovirus, HFMD, 2C protein, IFN-β, MDA5, RIG-I

## Abstract

As a member of the enteroviruses, coxsackievirus A6 (CV-A6) has been a major cause of hand, foot, and mouth disease (HFMD) since 2008. It can infect both pediatric and adult populations, often leading to atypical HFMD. The host innate immune system plays a vital role in the development of enteroviral infections. However, the interplay between the host antiviral response and CV-A6 has not been well investigated. In the present study, we demonstrated that the 2C protein from CV-A6 (2C_CV-A6_) suppresses interferon beta (IFN-β) production in HEK293T cells. Further results indicated that 2C_CV-A6_ interacts with both melanoma differentiation-associated gene 5 (MDA5) and retinoic acid-inducible gene I (RIG-I) and induces the degradation of these RNA sensors through proteases in the lysosomal pathway. This function also applies to 2C proteins from enterovirus A71 (2C_EV-A71_) and coxsackievirus B3 (2C_CV-B3_) but not CV-A16 2C (2C_CV-A16_) for its incompetence in MDA5 and RIG-I recognition. Partial depletion and amino acid substitution analyses indicated that the F28A, V75A, and I96V mutations significantly compromised 2C_CV-A6_-induced MDA5/RIG-I depletion. Surprisingly, unlike V75A and I96V that interrupt the 2C_CV-A6_-MDA5/RIG-I interaction, 2C_CV-A6_ F28A remained competent in MDA5/RIG-I binding, suggesting that the interaction alone is not sufficient for 2C-mediated reduction. Additional tests indicated that CV-A6 viruses containing the 2C F28A mutation were less efficient in IFN-β suppression, which is associated with compromised viral replication and release in infected rhabdomyosarcoma (RD) cells, suggesting that 2C-mediated immune regulation plays a vital role in enteroviral replication. Taken together, our data reveal a novel mechanism by which enteroviral 2C proteins antagonize the host innate antiviral immune response.

**IMPORTANCE:**

Coxsackievirus A6 (CV-A6) is a major emerging pathogen associated with atypical hand, foot, and mouth disease and can cause serious complications such as encephalitis, acute flaccid paralysis, and neurorespiratory syndrome. Therefore, revealing the associated pathogenic mechanisms could benefit the control of CV-A6 infections. In this study, we demonstrate that the nonstructural 2C_CV-A6_ suppresses IFN-β production, which supports CV-A6 infection. This is achieved by depleting RNA sensors such as melanoma differentiation-associated gene 5 and retinoic acid-inducible gene I (RIG-I) through the lysosomal pathway. Such a function is shared by 2C_EV-A71_ and 2C_CV-B3_ but not 2C_CV-A16_, suggesting the latter might have an alternative way to promote viral replication. This study broadens our understanding of enterovirus 2C protein regulation of the RIG-I-like receptor signaling pathway and reveals a novel mechanism by which CV-A6 and other enteroviruses evade the host innate immune response. These findings on 2C may provide new therapeutic targets for the development of effective inhibitors against CV-A6 and other enterovirus infections.

## INTRODUCTION

Hand, foot, and mouth disease (HFMD) caused by various enteroviruses is common in <5-year-old children throughout the world, particularly in the Asia-Pacific region (1). Among human enteroviruses, enterovirus A71 (EV-A71) and coxsackievirus A16 (CV-A16) are the most reported HFMD-causing pathogens. However, since 2008, HFMD outbreaks caused by coxsackievirus A6 (CV-A6) and A10 (CV-A10) have been frequently reported in Europe (2), Japan (3), and some developed regions in China (4). Several outbreaks of CV-A6-associated HFMD have occurred in China since 2013 (5 – 8), and the virus has emerged as the predominant enterovirus serotype in Shenzhen, China (9).

Similar to other enteroviruses, CV-A6 has a single-stranded positive-sense RNA genome with a length of ~7,400 nucleotides. The genome contains a single open reading frame that encodes a polyprotein that can self-digest into four structural (VP1, VP2, VP3, and VP4) and seven nonstructural proteins (2A, 2B, 2C, 3A, 3B, 3C, and 3D) (10, 11). Containing 329 amino acid residues, 2C is one of the most conserved and complex nonstructural protein and plays a vital role in the enteroviral life cycle (12). It comprises an adenosine triphosphatase (ATPase) domain, a zinc finger structure, and an alpha helix at the end of its C-terminal region (13). Several studies have previously indicated that enterovirus 2C plays crucial roles in host cell membrane rearrangements (14 – 17), RNA replication (18 – 21), viral infection, viral replication (22), encapsidation (23, 24), morphogenesis (25 – 27), immune regulation, and ATPase (28, 29), helicase (28, 30, 31), and chaperone activities (22, 32).

The innate immune system is the first line of host defense against viral infections and is linked to the activation and programming of adaptive immune responses (33). The type I IFN production pathway is activated by pattern recognition receptors that sense pathogen-associated molecular patterns to antagonize viral invasion (34, 35). Melanoma differentiation-associated gene 5 (MDA5) and retinoic acid-inducible gene I (RIG-I) are two major RNA sensors in human cells (36). Both contain a central DExD/H box ATPase/helicase domain that recognizes viral RNA, two N-terminal caspase activation and recruitment domains (CARDs), and a C-terminal regulatory/repression domain (37). Upon viral RNA infection, MDA5 and RIG-I undergo conformational alterations and interact with the adaptor mitochondrial antiviral signaling protein (MAVS, also known as IPS-1, VISA, or Cardif) through their CARD domain. As a result, a complex containing the TRAF3- and IκB-related kinases TBK1 and IKK*ε* is recruited by activated MAVS, which in turn directly phosphorylates IFN-regulatory factor 3 (IRF3) and IRF7. Phosphorylated IRF3 and IRF7 then translocate from the cytoplasm to the nucleus to induce type I IFN production, interferon-stimulated genes (ISGs), and inflammatory responses (38 – 42). Accordingly, these RIG-I-like receptor (RLR)-sensing pathways play an important role in activating the IFN signaling pathway.

Although the production of type I IFN elevates the cellular antiviral state against RNA viral infection, many enteroviruses, such as CV-A6, EV-A71, CV-A16, and EV-D68, have evolved strategies to inhibit IFN activation pathways (43, 44). Nonstructural 2C proteins from enteroviruses have also been shown to regulate the NF-κB pathway for viral replication and cell survival to evade host immune responses (45 – 47). However, little is known about the function of enteroviral 2C proteins in regulating the RLR pathway.

In the current study, we demonstrated that the 2C protein from CV-A6 (2C_CV-A6_) efficiently reduced IFN-β production in HEK293T cells by suppressing the activity of the *IFNB* promoter. Further tests showed that 2C_CV-A6_ interacted with both MDA5 and RIG-I and induced their degradation via proteases in the lysosomal pathway. Tests using 2C_CV-A6_ mutations suggested a correlation between 2C_CV-A6_-mediated RIG-I/MDA5 reduction and 2C_CV-A6_-triggered IFN-β reduction. Interestingly, MDA5 and RIG-I were recognized by 2C_EV-A71_ and 2C_CV-B3_ but not by 2C_CV-A16_, indicating that repression of the RLR pathway might be an evolutionary feature of enteroviral 2C proteins. Finally, we determined that amino acid F28 is critical for 2C_CV-A6_ to reduce the protein levels of RIG/MDA5 and subsequent production of IFN-β, which plays a crucial role in regulating CV-A6 replication. Collectively, these results illustrate the importance of enteroviral 2C proteins in regulating RLR-sensing pathways and provide novel insights into enteroviral regulation of host antiviral responses.

## RESULTS

### 2C protein of CV-A6 blocks IFN-β production

**Fig 1:**
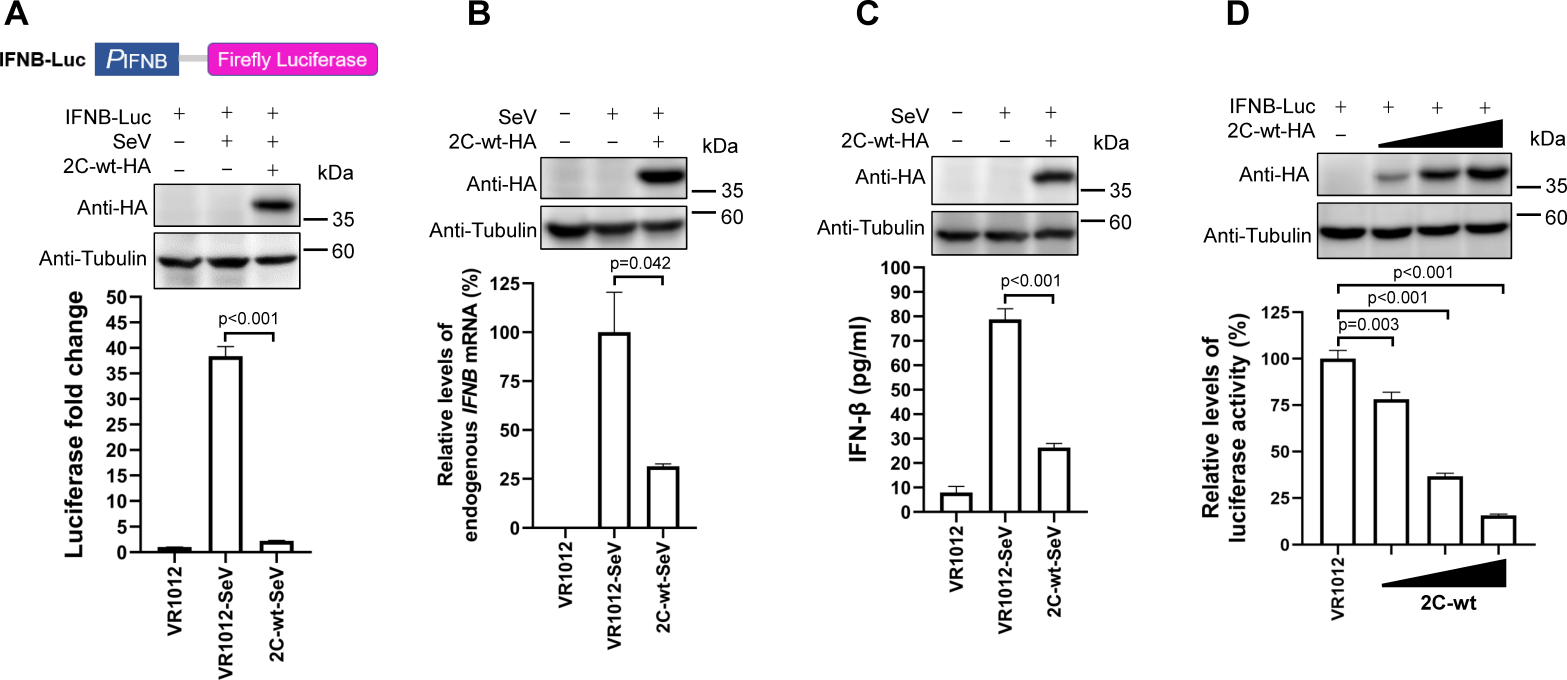
2C_CV-A6_ blocks the production of IFN-β. (**A**) Schematic of pGL3-IFNB-Luc vector. HEK293T cells were co-transfected with IFNB-Luc and 2C-wt-HA or VR1012 plasmid. Twenty-four hours after transfection, cells were left untreated or infected with SeV [20 hemagglutination (HA) units/mL] for 12 h, and the cell lysates were then assayed for luciferase activity. The expression levels of 2C protein were confirmed by western blot with an anti-HA antibody. (**B and C**) HEK293T cells were transfected with 2C-wt-HA or corresponding amounts of empty vector. Twenty-four hours after transfection, cells were infected with SeV (20 HA units/mL) for 12 h or were left uninfected, and were then collected to extract total RNA. The expression levels of endogenous *IFNB* mRNA were analyzed via quantitative reverse transcription PCR (**B**). The culture medium was harvested to detect IFN-β secretion using enzyme-linked immunosorbent assay (**C**). (**D**) HEK293T cells were co-transfected with IFNB-Luc (200 ng) and increasing amounts of plasmid 2C-wt-HA (50, 150, or 450 ng) or empty vector. Forty-eight hours after transfection, cells were collected for luciferase activity assay. Tubulin was used as a loading control in all western blot assays.

It is well known that the expression of interferons in mammalian cell lines can be stimulated by the RNA virus Sendai virus (SeV) (48, 49). To examine whether 2C_CV-A6_ can antagonize IFN-β production, HEK293T cells were co-transfected with the reporter plasmid IFNB-Luc and the plasmid encoding 2C_CV-A6_ or an empty vector VR1012. At 24 h post-transfection, the cells were mock infected or infected with SeV for 12 h. As shown in Fig. 1A, expression of 2C_CV-A6_ significantly inhibited SeV-induced *IFNB* promoter activity. Additional experiments were performed without IFNB-Luc transfection, and the levels of endogenous *IFNB* mRNA and secreted IFN-β were examined. SeV enhanced the endogenous levels of *IFNB* mRNA and the extracellular levels of IFN-β, which were significantly reduced by 2C_CV-A6_ expression (Fig. 1B and C). We then tested 2C_CV-A6_’s effects on *IFNB* promoter activity in HEK293T cells without SeV infection. As shown in Fig. 1D, the *IFNB* promoter activity was repressed by the presence of 2C_CV-A6_ in a dose-dependent manner. Collectively, these results suggest that the CV-A6 2C protein regulates IFN-β production via regulation of the *IFNB* promoter.

### CV-A6 2C mediates the degradation of MDA5 and RIG-I

**Fig 2:**
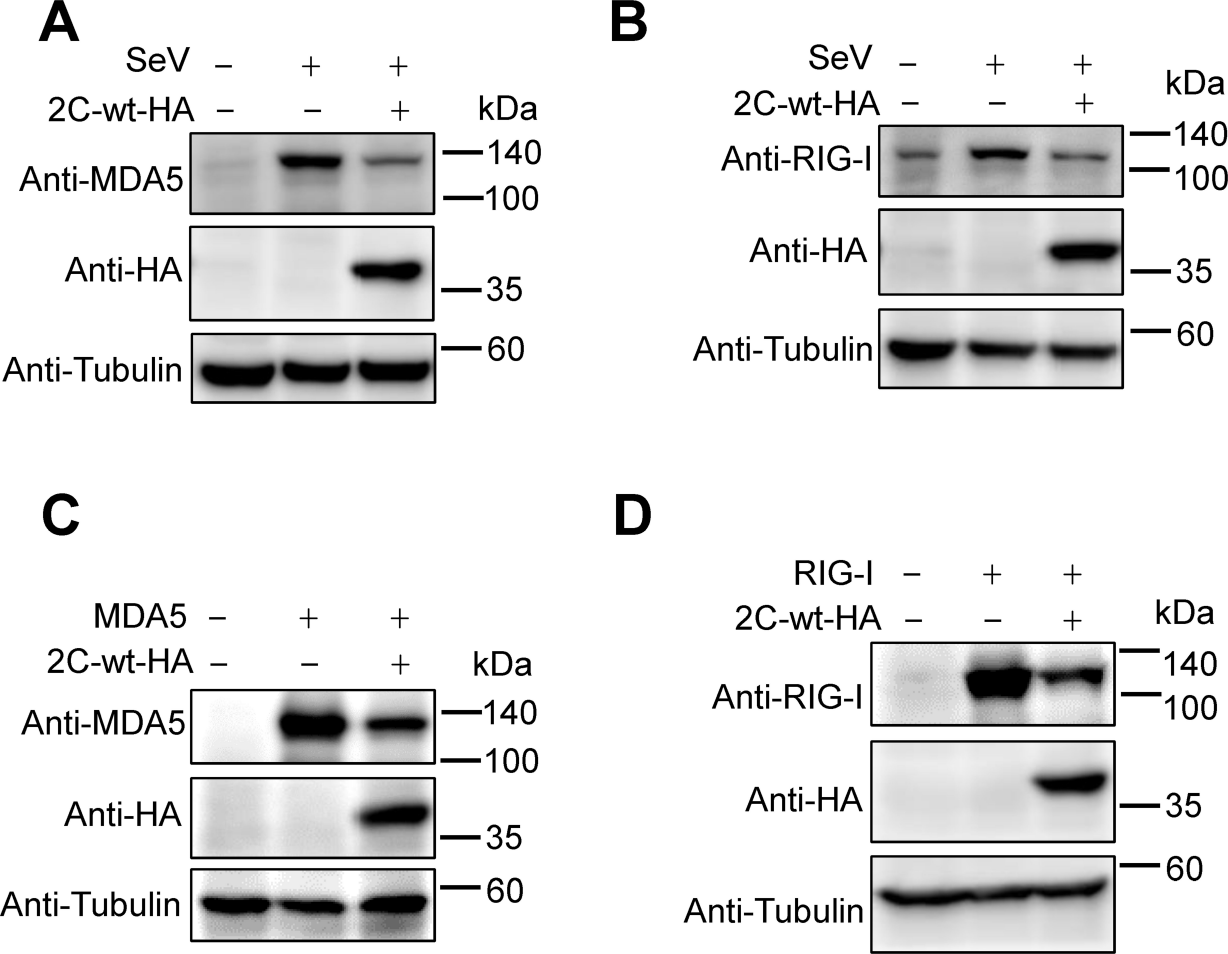
2C_CV-A6_ degrades both exogenous and endogenous MDA5 and RIG-I proteins. (**A and B**) HEK293T cells were co-transfected with 2C-wt-HA and MDA5-no-tag (**A**) or RIG-I-no-tag (**B**) expression plasmids. After 48 h, cells were collected for western blotting using the indicated antibodies. (**C and D**) HEK293T cells were transfected with 2C-wt-HA or VR1012 empty vector. Twenty-four hours after transfection, cells were infected with SeV (20 HA units/mL) for 12 h or left uninfected, and then collected for western blotting using the indicated antibodies. Tubulin was used as a loading control in all western blot assays.

SeV is a strong inducer of RLR-mediated IFN-β signaling (50). MDA5 and RIG-I belong to the RLR family and are considered the most important cytosolic viral RNA sensors (51). Interestingly, in addition to acting as sensors for detecting exogenous viral RNA, both MDA5 and RIG-I are induced by IFN activation (52). Consistently, by triggering IFN expression, SeV infection also increased the endogenous levels of MDA5 and RIG-I proteins (Fig. 2A and B). However, the expression of 2C_CV-A6_ induced the depletion of MDA5 and RIG-I, suggesting that these two sensors are targets of 2C_CV-A6_.

To confirm this hypothesis, HEK293T cells were co-transfected with an expression plasmid encoding full-length MDA5 or RIG-I, along with CV-A6-2C-wt-HA or an empty vector. As shown in Fig. 2C and D, overexpression of 2C_CV-A6_ resulted in significant degradation of either MDA5 or RIG-I proteins. Thus, 2C_CV-A6_ suppresses the host IFN signaling system through destabilization of MDA5 and RIG-I.

### CV-A6 2C protein reduces the expression of MDA5 and RIG-I via the proteases in the lysosomal pathway

**Fig 3:**
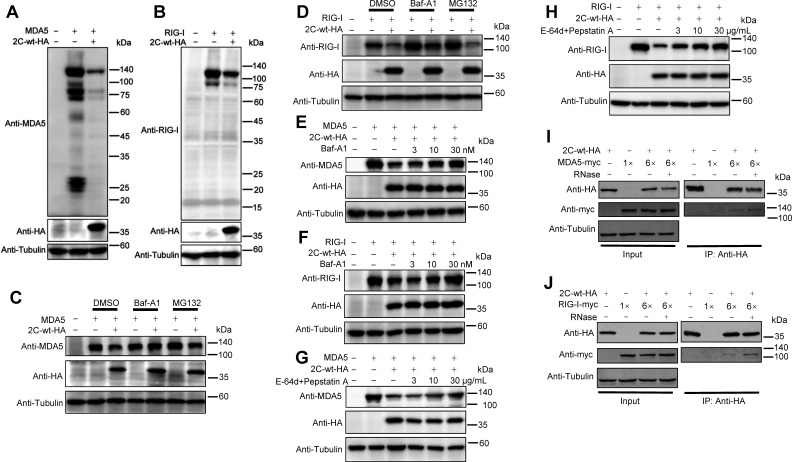
2C_CV-A6_ protein reduces the expression levels of MDA5 and RIG-I through the proteases in the lysosomal pathway. (**A and B**) HEK293T cells were transfected with 2C-wt-HA, together with MDA5-no-tag (**A**) or RIG-I-no-tag (**B**) plasmids. Forty-eight hours after transfection, the cells were subjected to western blotting using the indicated antibodies. The entire membrane was visualized to detect potential digestion products of MDA5 or RIG-I. The multi-band phenomena detected for MDA5 in panel A is similar to previous reported observations (56, 57). (**C and D**) VR1012-2C-wt-HA and MDA5-no-tag (**C**) or RIG-I-no-tag (**D**) plasmids were co-transfected into HEK293T cells for 24 h and then treated with either the proteasomal inhibitor MG132 (20 µM), the autophagy-lysosome inhibitor Baf-A1 (10 nM), or dimethyl sulfoxide (DMSO) (vehicle control) for a further 16 h. Whole-cell lysates were prepared for western blot analysis with the indicated antibodies. (**E and F**) HEK293T cells were transfected with plasmids to express MDA5-no-tag (**E**) or RIG-I-no-tag (**F**) alone, or co-transfected with a plasmid-expressing 2C-wt-HA for 24 h, and then treated with increasing amounts of Baf-A1 (3, 10, or 30 nM) for another 16 h. Whole-cell lysates were prepared 40 h post-transfection for western blot analyses with the indicated antibodies. (**G and H**) HEK293T cells were transfected as in E and F. Twenty-four hours post-transfection, the cells were then treated with increasing amounts of E-64d and Pepstatin A (3, 10, or 30 µg/mL) for another 12 h. Whole-cell lysates were prepared 36 h post-transfection for western blot analyses with the indicated antibodies. (**I and J**) HEK293T cells in six-well plates were co-transfected with 2C-wt-HA and MDA5-myc (**I**) or RIG-I-myc (**J**), or transfected with 2C-wt-HA, MDA5, or RIG-I plasmids alone. Forty-eight hours after transfection, cells were harvested, and the cell lysates were treated with RNase or left untreated and incubated with anti-HA beads at 4°C overnight. Protein expression was detected by western blotting using the indicated antibodies. The expression of tubulin was used as a protein control in all western blot analyses.

The ubiquitin-proteasome and lysosome pathways are the two main routes for protein and organelle clearance in eukaryotic cells (53, 54). Saeed et al. reported the first comprehensive view of host protein digestion in cells infected with five representative enteroviruses, revealing enteroviral mechanisms of immune evasion, resource exploitation, and pathogenesis (55). To further investigate the mechanism underlying 2C-mediated MDA5 and RIG-I depletion, HEK293T cells were transfected with CV-A6 2C and MDA5 or RIG-I expression plasmids and subjected to western blotting. As shown in Fig. 3A and B, no obvious increase in the smaller band was detected for MDA5 or RIG-I in the presence of 2C_CV-A6_, suggesting that the 2C_CV-A6_-mediated MDA5 and RIG-I reduction was not due to 2C_CV-A6_-induced protein cleavage.

MG132, an inhibitor of proteasome-mediated proteolysis, and Baf-A1, an inhibitor of the autosome-lysosome pathway, were tested for their effects on 2C_CV-A6_-mediated MDA5 and RIG-I degradation. Western blotting showed that Baf-A1, but not MG132, rescued both MDA5 and RIG-I from 2C_CV-A6_-induced depletion (Fig. 3C and D), which was further confirmed using Baf-A1 at different concentrations (Fig. 3E and F). We tested two additional lysosomal protease inhibitors, E-64d and Pepstatin A, in the degradation experiment. As shown in Fig. 3G and H, the degradation of MDA5 and RIG-I by 2C-wt could be restored by the addition of E-64d and Pepstatin A in a dose-dependent manner. Taken together, these results revealed that 2C_CV-A6_ plays a key role in preventing MDA5 or RIG-I degradation by proteases in the lysosomal pathway.

Target protein interaction is essential for the autosome-lysosome pathway (58); thus, we tested whether 2C_CV-A6_ could interact with MDA5 or RIG-I using co-immunoprecipitation (co-IP) assays. Western blotting of the eluted samples showed that both MDA5 and RIG-I immunoprecipitated with HA-tagged 2C_CV-A6_, whereas no MDA5 or RIG-I was pulled down in the negative control group (Fig. 3I and J). Moreover, the interaction of MDA5 or RIG-I with 2C_CV-A6_ was not disrupted by RNase treatment, indicating that the specific interaction between 2C_CV-A6_ and MDA5 or RIG-I was RNA independent (Fig. 3I and J). These results indicate that 2C_CV-A6_ interacts with MDA5 and RIG-I and induces their depletion via proteases in the lysosomal pathway.

### Different enterovirus 2C proteins distinctively regulate MDA5/RIG-I stability and *IFNB* promoter activity

**Fig 4:**
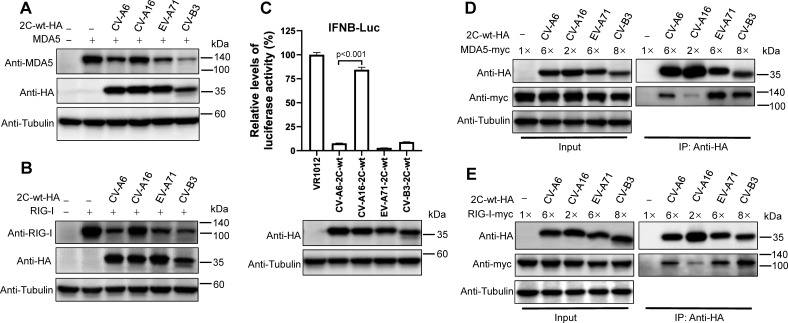
Effects of multiple enteroviral 2C proteins on MDA5 and RIG-I degradation, and IFNB promoter activity. (**A and B**) Four enterovirus 2C-wt-HA expression plasmids were co-transfected with MDA5-no-tag (**A**) or RIG-I-no-tag (**B**) into HEK293T cells for 48 h. The cells were collected and subjected to western blot assays using the indicated antibodies. (**C**) HEK293T cells were co-transfected with IFNB-Luc plasmid, together with four wild-type enterovirus 2C expression plasmids for 48 h; the cells were then subjected to a luciferase activity assay. The expression levels of 2C proteins were detected by western blotting with anti-HA and anti-tubulin antibodies. (**D and E**) HEK293T cells in six-well plates were transfected with plasmids encoding MDA5-myc (**D**) or RIG-I-myc (**E**), together with one of four 2C-wt-HA expression plasmids. Forty-eight hours later, cells were harvested, and cell lysates were incubated with anti-HA beads at 4°C overnight. The input and eluted samples were detected by western blotting using the indicated antibodies, with tubulin as a loading control.

As the enteroviral 2C protein is one of the most conserved and complex nonstructural protein (12), it is reasonable to speculate that other enteroviral 2C proteins also degrade MDA5 and RIG-I and consequently suppress *IFNB* promoter activity. To test this hypothesis, we first constructed three additional plasmids expressing 2C proteins from other prevalent enteroviruses, namely CV-A16, EV-A71, and CV-B3. As shown in Fig. 4A and B, similar to 2C_CV-A6_, the 2C proteins from EV-A71 (2C_EV-A71_) and CV-B3 (2C_CV-B3_) degraded both MDA5 and RIG-I, whereas the 2C protein from CV-A16 (2C_CV-A16_) did not. Further tests indicated that 2C_EV-A71_ and 2C_CV-B3_, but not 2C_CV-A16_, significantly suppressed *IFNB* promoter-driven luciferase expression in HEK293T cells (Fig. 4C). This finding validated our previous hypothesis that 2C regulates the IFN signaling pathway through MDA5/RIG-I depletion. We then investigated why CV-A16 2C failed to deplete MDA5/RIG-I and tested its interaction with these two sensors through co-IP experiments. As expected, 2C_CV-A16_ almost lost its ability to interact with MDA5 or RIG-I, whereas 2C_EV-A71_ and 2C_CV-B3_ were fully capable of interacting (Fig. 4D and E). Such a failure in MDA5/RIG-I depletion may indicate an evolutionary divergence of 2C_CV-A16_; in other words, 2C_CV-A16_ may have evolved to perform an alternative function that promotes viral replication. These results indicate that MDA5/RIG-I recognition is necessary, but not sufficient, for enteroviral 2C-mediated degradation.

### Positions 75 and 96 are essential for enteroviral 2C to recognize MDA5 and RIG-I

**Fig 5:**
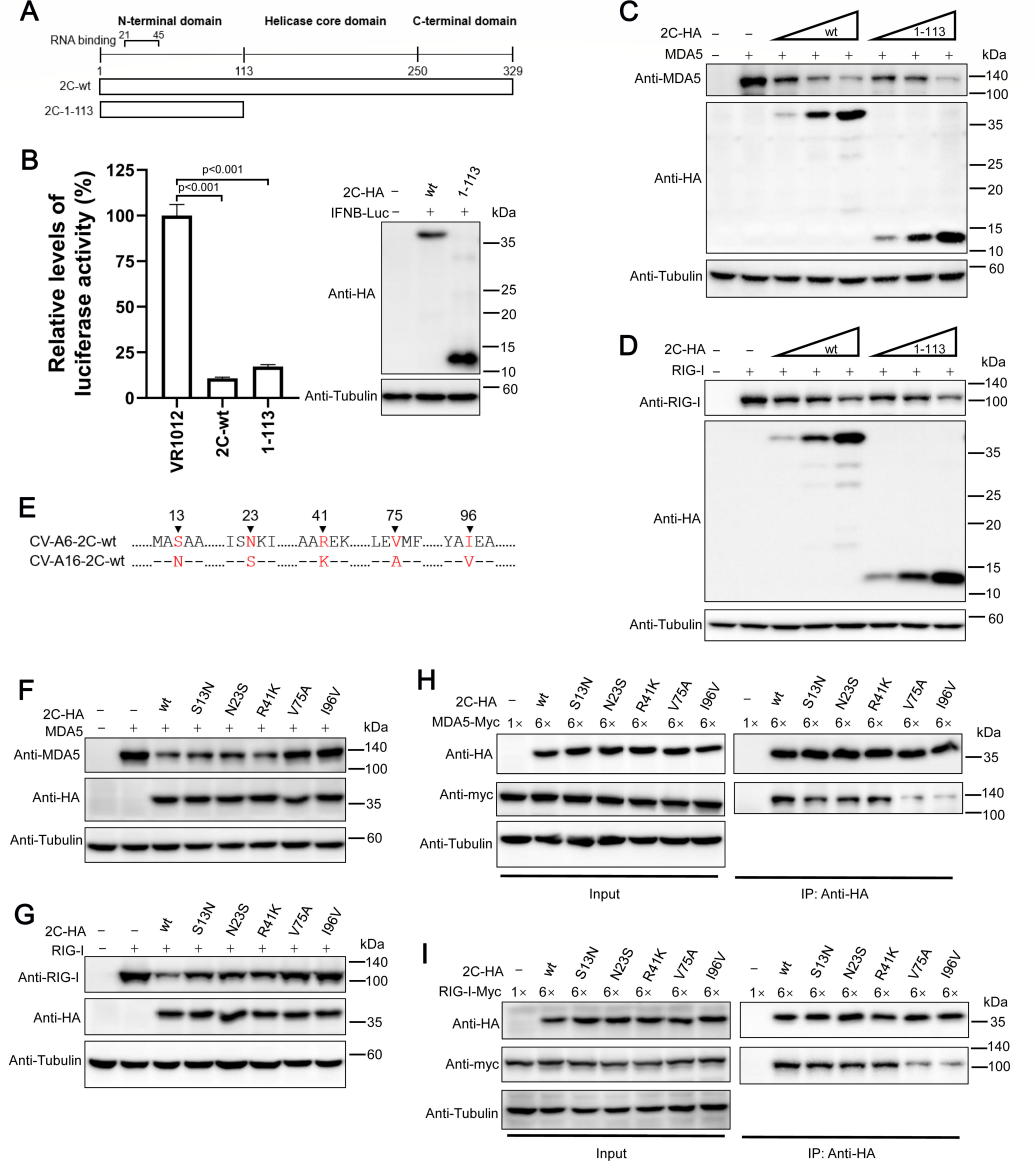
Amino acids V75 and I96 of 2C_CV-A6_ are crucial for the interaction with and degradation of MDA5 and RIG-I. (**A**) Schematic of 2C_CV-A6_ N-terminal truncation constructs. (**B**) IFNB-Luc and 2C-wt-HA or the truncation mutant were co-transfected into HEK293T cells; the cells were then subjected to luciferase assays at 48 h post-transfection. The expression of 2C-wt and truncated protein was confirmed by western blotting with an anti-HA antibody. (**C and D**) MDA5 (**C**) or RIG-I (**D**) was co-transfected with wild-type or five mutated 2C-HA expression constructs into 24-well plates of HEK293T cells. Forty-eight hours post-transfection, cells were harvested for measurement of MDA5 and RIG-I expression by western blot analyses. (**E**) Sequence alignment of 1–113 fragment of 2C_CV-A6_ and 2C_CV-A16_. (**F and G**) Wild-type or five mutated CV-A6-2C-HA expression plasmids were co-transfected with MDA5-no-tag (**F**) or RIG-I-no-tag (**G**) into HEK293T cells for 48 h. The cells were collected and subjected to western blot assay using the indicated antibodies. (**H and I**) HEK293T cells were co-transfected in six-well plates with MDA5-myc (**H**) or RIG-I-myc (**I**), together with wild-type or five mutated CV-A6-2C-HA expression plasmids, as indicated, and were harvested at 48 h post-transfection. The cells were then subjected to an immunoprecipitation assay with anti-HA beads. Input and eluted samples were detected by western blotting. Tubulin was used as a loading control.

The 2C protein typically contains an N-terminal membrane-binding domain, central ATPase domain, cysteine-rich domain, and C-terminal helical domain (22, 59) (Fig. 5A). In 2018, Li et al. reported that amino acids 1–125 at the N-terminus of the 2C protein of EV-A71 are sufficient for APOBEC3G (A3G) degradation, whereas removing internal amino acids 26–40 abolishes the function of 2C_EV-A71_ (60). To map the minimal functional region of 2C responsible for MDA5 and RIG-I degradation, a C-terminal truncation was first performed on 2C_CV-A6_ (Fig. 5A). Luciferase assay results showed that the N-terminal 1–113 fragment of 2C_CV-A6_ was sufficient for *IFNB* promoter regulation (Fig. 5B), suggesting that this fragment contains essential residues for MDA5/RIG-I degradation. As expected, 2C_CV-A6_ 1–113 efficiently reduced MDA5 and RIG-I expression (Fig. 5C and D).

It was then easy to speculate that the differences in this region (1–113) might explain why 2C_CV-A16_ failed to trigger MDA5/RIG-I depletion. Protein sequence alignment showed that 2C_CV-A6_ and 2C_CV-A16_ share different amino acid residues at positions 13, 23, 41, 75, and 96 in this region (Fig. 5E). Point mutations were then introduced in 2C_CV-A6_ by replacing these residues with the corresponding residues from 2C_CV-A16_. Subsequent test results suggested that although mutations such as S13N, N23S, and R41K mildly disrupted the 2C_CV-A6_-mediated MDA5/RIG-I reduction, V75A and I96V significantly compromised 2C_CV-A6_’s ability to deplete MDA5/RIG-I (Fig. 5F and G). Consistently, both V75A and I96V disrupted the interaction between 2C_CV-A6_ and MDA5/RIG-I (Fig. 5H and I). These data indicate that these two residues are essential for MDA5/RIG-I recognition, explaining 2C_CV-A16_’s inefficient MDA5/RIG-I binding and reduction.

### Position F28 is important for 2C-mediated MDA5/RIG-I depletion but not for MDA5 or RIG-I-2C interaction

**Fig 6:**
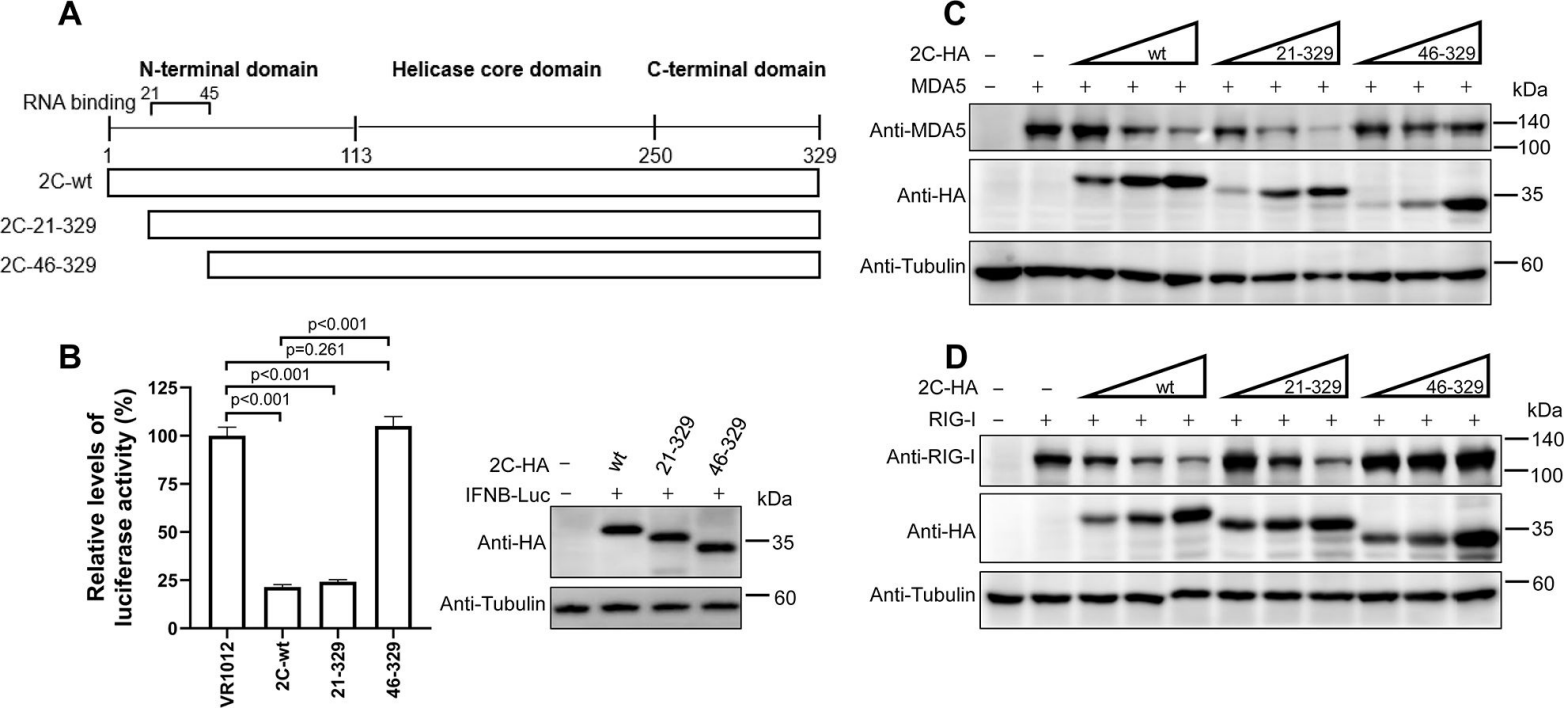
Amino acids 21–45 of the 2C_CV-A6_ N-terminus are required for MDA5 and RIG-I degradation. (**A**) Schematic of 2C_CV-A6_ truncation constructs. (**B**) IFNB-Luc and 2C-wt-HA or the truncation mutants were co-transfected into HEK293T cells; the cells were then subjected to luciferase assays at 48 h post-transfection. The expression of 2C-wt and truncated proteins was confirmed by western blotting with an anti-HA antibody. (**C and D**) HEK293T cells were co-transfected with MDA5-no-tag (C, 500 ng) or RIG-I-no-tag (D, 500 ng) plasmid, together with increasing amounts of plasmid 2C-wt-HA (50, 150, or 450 ng), 2C-21-329-HA (100, 300, or 900 ng), and 2C-46-329-HA (50, 150, or 450 ng) or VR1012 empty vector. Forty-eight hours post-transfection, cells were collected for western blotting with the indicated antibodies. In all western blots, tubulin was used as a protein loading control.

To further verify the importance of the N-terminal fragment in 2C-mediated MDA5/RIG-I degradation, two N-terminal truncations were performed in 2C_CV-A6_ (Fig. 6A). Initial tests indicated that 2C_CV-A6_ 21–329 remained effective in *IFNB* promoter repression, while 2C_CV-A6_ 46–329 no longer reduced *IFNB* promoter activity (Fig. 6B). Further tests confirmed that 2C_CV-A6_ 46–329, but not 2C_CV-A6_ 21–329, could no longer induce the depletion of MDA5 or RIG-I (Fig. 6C and D). These findings correlate 2C_CV-A6_-mediated MDA5/RIG-I degradation with 2C_CV-A6_-induced innate immune repression, suggesting that fragment 21–45 is important for 2C_CV-A6_ to trigger MDA5/RIG-I depletion.

**Fig 7:**
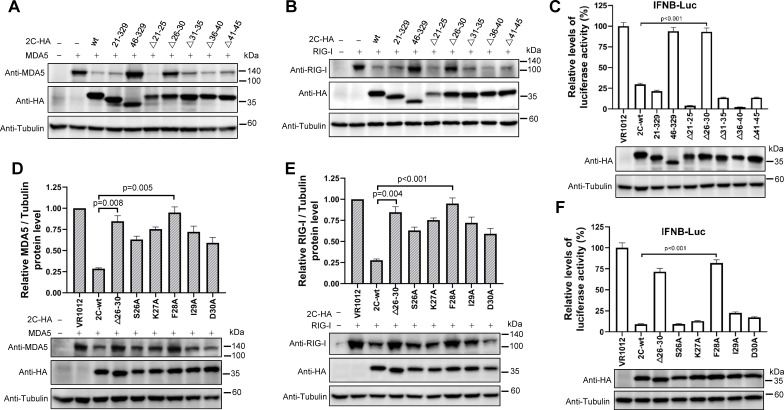
Position F28 is important for 2C_CV-A6_-mediated MDA5/RIG-I depletion and loss of the ability to inhibit *IFNB* promoter activity. (A, B, D, and E) MDA5 (**A and D**) or RIG-I (**B and E**) was co-transfected with wild-type, truncated, and mutated 2C-HA expression constructs into HEK293T cells. Forty-eight hours later, cells were harvested for measurement of MDA5 and RIG-I expression by western blot analysis. ImageJ software (NIH) was used to quantitate protein band intensities, and the value of MDA5 (**D**) or RIG-I (**E**) in the absence of 2C was set to 1. The data shown are calculated based on results from three independent experiments. (**C and F**) HEK293T cells were co-transfected with IFNB-Luc, 2C-wt-HA, truncation, or mutation expression plasmids. Forty-eight hours post-transfection, the cells were collected for luciferase activity determination. The expression of 2C-wt, truncated, or mutated proteins was detected via western blotting with an anti-HA antibody. Tubulin was used as a loading control.

To further examine the N-terminal region at higher resolution, five serial truncation plasmids between amino acids 21 and 45 of 2C were generated and tested. Western blotting and luciferase assay results showed that the 2C_CV-A6_ Δ21–25, Δ31–35, Δ36–40, and Δ41–45 mutants remained effective in inducing MDA5/RIG-I degradation and inhibiting *IFNB* promoter activity, whereas Δ26–30 did not (Fig. 7A through C). Subsequently, single alanine substitution mutants within amino acids 26–30 of 2C were constructed and tested. Western blotting and luciferase assay results revealed that F28A, but not the other amino acid substitutions, significantly compromised the ability of 2C_CV-A6_ to degrade MDA5 and RIG-I (Fig. 7D and E). Consequently, the same mutation reduced 2C_CV-A6_’s efficiency of repressing *IFNB* promoter activity (Fig. 7F). Taken together, these results indicate that amino acid F28 plays an important role in the 2C_CV-A6_-mediated regulation of RLR signaling pathways.

**Fig 8:**
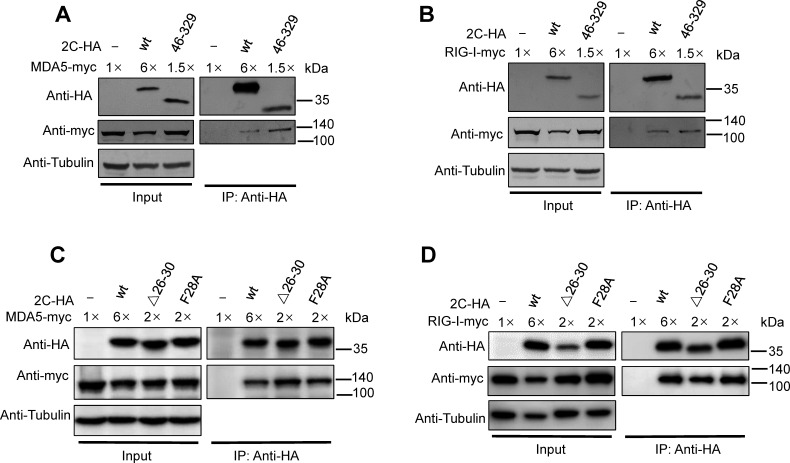
Truncated and mutant 2C_CV-A6_, which have lost the ability to degrade MDA5 and RIG-I, still interact with MDA5 and RIG-I. HEK293T cells were co-transfected with MDA5-myc (**A and C**) or RIG-I-myc (**B and D**), together with wild-type, truncated, or mutated 2C-HA expression plasmids, as indicated, and were harvested 48 h post-transfection. The cells were then subjected to an immunoprecipitation assay with anti-HA beads. Input and eluted samples were detected by western blotting. Tubulin was used as a loading control.

We then determined why the above 2C_CV-A6_ mutants (i.e., 46–329, Δ26–30, and F28A) failed to deplete MDA5/RIG-I. Myc-tagged MDA5 or RIG-I expression plasmids were co-transfected into HEK293T cells with a vector-expressing wild-type 2C_CV-A6_ or one of the above mutants. Exogenously expressed wild-type 2C_CV-A6_ or its mutants were isolated through co-IP experiments, and bound MDA5-myc or RIG-I-myc was detected by western blotting. Surprisingly, as shown in Fig. 8, all the tested mutants (i.e., 46–329, Δ26–30, and F28A) remained effective in MDA5/RIG-I recognition. Notably, these data correlated with the fact that all the tested mutants contained V75 and I96, which have been confirmed to be essential for MDA5/RIG-I binding (Fig. 5H and I). Altogether, this suggests that F28 is not involved in target protein interactions but might be crucial for 2C_CV-A6_ to introduce target proteins to the subsequent lysosomal pathway for degradation.

### 2C-mediated MDA5/RIG-I depletion plays an important role in CV-A6 replication

**Fig 9:**
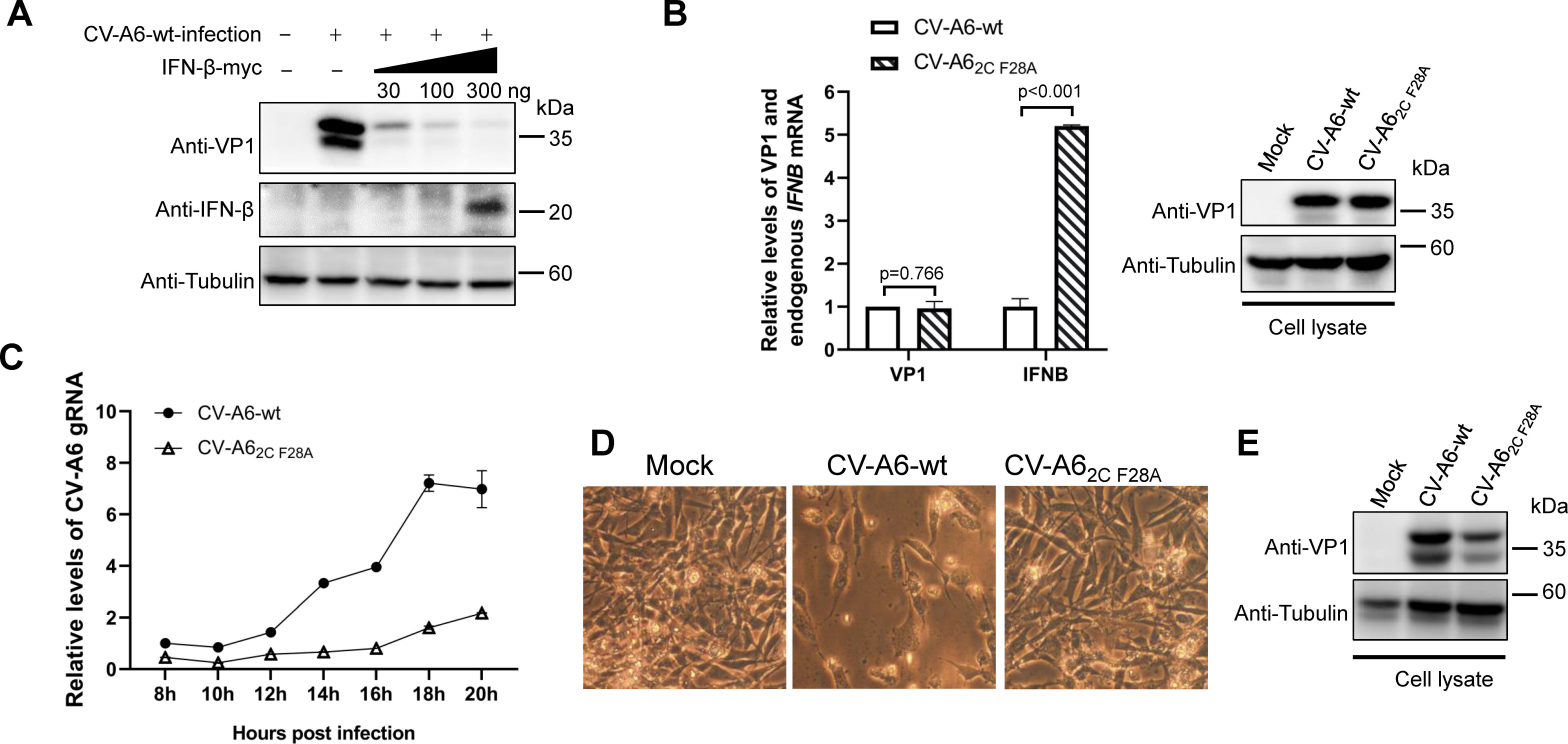
2C_CV-A6_ plays an important role in IFN-β regulation and viral replication during CV-A6 infection. (**A**) RD cells were transfected with increasing amounts (30, 100, or 300 ng) of VR-IFN-β-myc plasmid in 24-well plates. Twenty-four hours after transfection, the cells were infected with equal amounts of CV-A6 recombinant virus for another 48 h. Cell lysates were collected for western blot analysis using the indicated antibodies. Tubulin antibody was used as a protein-loading control. (**B**) RD cells in 24-well plates were infected with adjusted amounts of CV-A6- and CV-A6_2C F28A_-rescued viruses to achieve equal infection with both viruses. Twenty-four hours post-infection, the cells were collected. Half of the whole-cell lysate was used to extract RNA for quantitative reverse transcription PCR (qRT-PCR) to detect VP1 mRNA levels, and the other half was subjected to western blotting to confirm CV-A6 VP1 expression. Both results were used to confirm equal infection with both viruses. Endogenous *IFNB* mRNA levels were detected using qRT-PCR, with levels of *ACTB* mRNA used as a loading control. (**C**) One-step growth curves of the CV-A6 and CV-A6_2C F28A_ recombinant viruses. Based on genome quantification by qRT-PCR, similar amounts of these viruses were used to infect RD cells. After 6 h of adsorption, the infected cells were washed once with PBS, the medium was replaced with maintenance medium, and the levels of released viruses in the medium were determined by qRT-PCR at 2-h intervals, starting at 8 h post-adsorption. (**D and E**) RD cells were infected with equal amounts of CV-A6 or CV-A6_2C F28A_ recombinant virus. At 72 h after infection, the cells were observed and photographed using a light microscope (**D**). Whole-cell lysates were collected for western blotting using an anti-CV-A6 VP1 antibody (**E**).

To confirm the effect of 2C-mediated MDA5/RIG-I depletion on enteroviral replication, we first tested whether altered IFN-β levels affect CV-A6 proliferation in RD cells. Different amounts of the VR-IFN-β-myc plasmid were transfected into RD cells, which were then infected with wild-type CV-A6 virus 24 h post-transfection. The cells were collected 48 h post-infection and subjected to western blotting. The results showed that ectopic expression of IFN-β significantly inhibited the replication of wild-type CV-A6 in a dose-dependent manner (Fig. 9A). We then determined whether the F28A mutation in 2C caused differences in endogenous IFN-β levels in CV-A6-infected cells. A proviral vector-expressing wild-type CV-A6 was produced as previously described (61) and used to generate a plasmid-expressing CV-A6 containing the F28A mutation in its 2C protein (CV-A6_2C F28A_). RD cells were then infected with rescued wild-type CV-A6 or CV-A6_2C F28A_, the dosages of which were carefully adjusted to achieve similar viral expression when the infected cells were collected 24 h post-infection (as indicated by both RNA and protein levels of VP1 in Fig. 9B). However, CV-A6_2C F28A_ triggered more *IFNB* transcription than CV-A6 (*P* < 0.001, Fig. 9B). This confirmed that 2C suppresses endogenous IFN-β production in CV-A6-infected cells.

These data also indicate that reducing endogenous IFN-β levels may contribute to the 2C-mediated promotion of enteroviral replication. To test this hypothesis, RD cells were infected with equal amounts of wild-type CV-A6 or CV-A6_2C F28A_, and a one-step growth assay was performed at 2-h intervals as previously described (62). As shown in Fig. 9C, the quantitative reverse transcription PCR (qRT-PCR) results targeting CV-A6 genomic RNA indicated that the levels of wild-type CV-A6 were consistently higher than those of CV-A6_2C F28A_ within 20 h of infection. Images after prolonged infection showed that wild-type CV-A6 caused cytopathogenic effect (CPE) in RD cells 72 h post-infection, which CV-A6_2C F28A_ failed to achieve (Fig. 9D). Western blotting of infected cells also confirmed that the viral replication efficiency was compromised in RD cells infected with CV-A6_2C F28A_ (Fig. 9E). These results indicate that the 2C-mediated suppression of RLR signaling pathways contributes to innate immune regulation during CV-A6 infection, providing an additional mechanism to enhance viral replication.

## DISCUSSION

**Fig 10:**
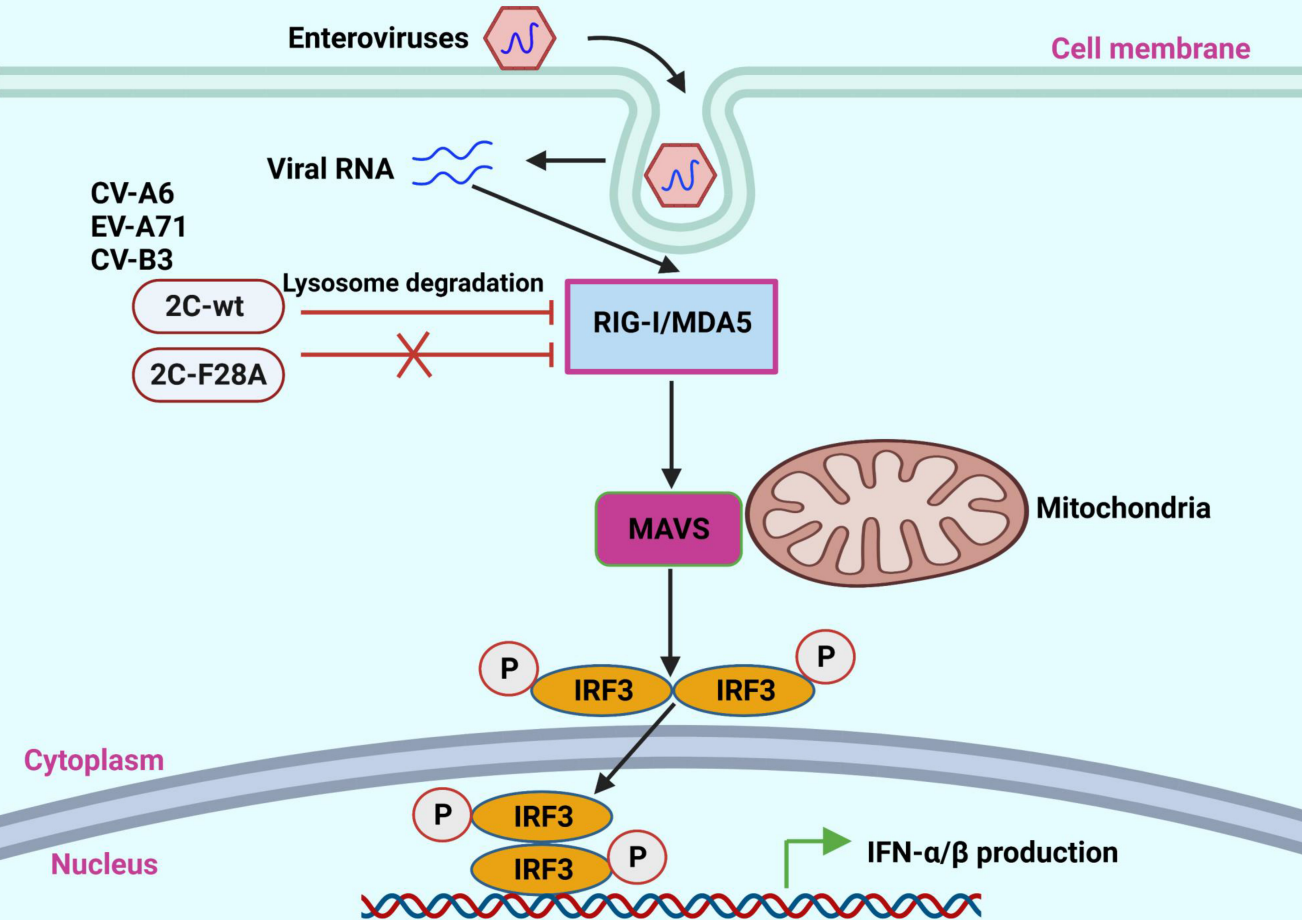
Proposed model of enterovirus 2C protein-mediated inhibition of MDA5/RIG-I signaling pathway. After enterovirus infection, the nonstructural 2C protein degrades MDA5 and RIG-I through the lysosomal pathway and thus inhibits activation of the *IFNB* promoter, ultimately blocking IFN-β production. However, F28A mutation compromises 2C’s ability to inhibit type I IFN signaling. This diagram was created with BioRender.com.

Generally, MDA5 and RIG-I are the main sensors of RNA viruses that trigger type I IFN production. Similar to RNA viruses, enteroviruses have evolved multiple mechanisms to suppress the RNA-sensing pathways in host cells. For instance, enteroviral nonstructural proteins such as 2A, 3C, and 3D induce dysfunction and/or destabilization of MDA5 and RIG-I (43, 56, 63, 64). In this study, we report that another enteroviral nonstructural protein, 2C from CV-A6, is also capable of triggering MDA5 and RIG-I depletion, resulting in a decrease in *IFNB* promoter activity and IFN-β production (Fig. 10). Subsequent results indicated that 2C_CV-A6_ interacts with MDA5 and RIG-I in an RNA-independent manner and induces their degradation via the proteases in the lysosomal pathway. Surprisingly, although 2C proteins from EV-A71 and CV-B3 were also capable of MDA5/RIG-I degradation, 2C from CV-A16 failed to do so because the latter did not recognize MDA5 or RIG-I properly. Additional tests on truncated 2C_CV-A6_ suggested that the N-terminal 21–45 amino acid fragment is important for MDA5/RIG-I removal, which was further narrowed down to one specific amino acid residue, F28. Interestingly, the F28A mutation did not affect the interaction between 2C and MDA5 or RIG-I, suggesting that this specific position plays an essential role in the transfer of MDA5/RIG-I to the lysosomal pathway. Consequently, introducing the F28A mutation into CV-A6 significantly elevated *IFNB* mRNA levels in CV-A6-infected RD cells, leading to a reduction in viral infectivity in both short- and long-term infections.

At first glance, it is puzzling that both MDA5 and RIG-I are downregulated by enteroviral 2C because, despite their structural and functional similarities, MDA5, but not RIG-I, has been reported to be critical for sensing enteroviral infection (65). In the presence of EV-A71 RNA, MDA5 triggers the activation of IRF3, which plays an important role in IFN activation (65). Therefore, it is reasonable for enteroviruses to counteract the function and/or stability of MDA5. However, it should be noted that in addition to being sensors, both MDA5 and RIG-I are effectors of the IFN signaling pathway (i.e., the genes coding for MDA5 and RIG-I are ISGs) (52). Moreover, introducing exogenous MDA5 or RIG-I alone can induce innate immune activation even in the absence of viral RNA (or other stimuli) (66, 67). Therefore, MDA5/RIG-I and IFN form a positive feedback loop during innate immune activation. Accordingly, depletion of both MDA5 and RIG-I would result in a lower level of IFN activation than the removal of either alone. This is not the first report on enteroviral proteins that suppress RIG-I. For example, EV-A71 infection can compromise RIG-I ubiquitination and consequently reduce IFN production (68). By removing both MDA5 and RIG-I, 2C inhibits IFN activation more effectively, which contributes to 2C’s critical role in enteroviral infection.

Surprisingly, the 2C protein from CV-A16, an enterovirus that often causes HFMD pandemics (5, 69 – 71), does not recognize MDA5/RIG-I and trigger their degradation. 2C_CV-A16_ and 2C_CV-A6_ share nine different amino acids at positions 13, 23, 41, 75, 96, 227, 271, 303, and 307. However, no single-point mutation at these sites could grant 2C_CV-A16_ the ability to destabilize MDA5 or RIG-I (data not shown), suggesting that complex cooperation among some or all these residues is essential for MDA5/RIG-I recognition and/or depletion. Indeed, during the revision of this manuscript, positions 75 and 96 on 2C_CV-A6_ and 2C_CV-A16_ were both determined crucial for MDA5/RIG-I binding and subsequent downregulation. Interestingly, for a 2C protein functional in MDA5/RIG-I degradation, the residues on positions 75 and 96 are not exclusively restricted. In fact, tested 2C_CV-A6_, 2C_EV-A71_, and 2C_CV-B3_ contain V75/I96, V75/T96, and Q75/A96, respectively, while all these three enteroviral proteins were capable of MDA5/RIG-I removal. Furthermore, it should be noticed that, removing MDA5/RIG-I might not be a conserved feature for all EV-A71, CV-A6, or CV-B3 2C proteins. For example, among 1,602 EV-A71 2C protein sequences retrieved from the NCBI Protein Database (https://www.ncbi.nlm.nih.gov/protein), 527 contain inactive residue A75. On the other hand, although most retrieved CV-A16 2C proteins are believed inactive in MDA5/RIG-I depletion as they contain A75 and V96, there are 4 out of 473 CV-A16 2C proteins that do have functional residues at both positions 75 and 96 and might be capable to trigger MDA5/RIG-I degradation. In general, it appears that positions 75 and 96 are probably under selection pressure, resulting in MDA5/RIG-I depletion as an evolutionarily selective instead of a conserved function for enteroviral 2C, which might provide possible explanations on the distinctive virulence observed for different enteroviral subtypes and even strains.

On the contrary, protein sequence alignment suggested that F28 is highly conserved among 2C proteins from different types of enteroviruses (99.78% or 3,211 out of 3,218 strains; data not shown). To our knowledge, F28, as a single amino acid residue, has not been revealed to be critical for any known function of the enteroviral 2C protein. Notably, the regions around F28 are essential for most of the known functions of 2C, including residues 1–38 for oligomerization, 21–45 for RNA binding, and 21–54 for membrane binding. These regions play important roles in supporting 2C in host cell membrane rearrangements, RNA replication, encapsidation, morphogenesis, and ATPase activity [reviewed in reference (32)]. Recently, Li et al. reported that fragment 1–53 is sufficient for EV-A71 2C-mediated APOBEC3G depletion, which in turn promotes viral replication (60). Meanwhile, fragment 1–113 of 2C_CV-A6_ is competent in reducing MDA5/RIG-I levels and subsequent IFN production, as revealed in this study. These findings indicate that blocking the N-terminal domain of enteroviral 2C may be an effective strategy to suppress enteroviral replication. Our data showed that F28A mutation alone significantly compromised the replication of CV-A6 (Fig. 9), further supporting the idea that enteroviral 2C is a promising target for the development of anti-enteroviral drugs.

## MATERIALS AND METHODS

### Cells, viruses, and reagents

HEK293T (no. CRL-11268) and human rhabdomyosarcoma (RD) cells (no. CCL-136) were obtained from the American Type Culture Collection (Manassas, VA, USA). Cells were grown in Dulbecco’s Modified Eagle’s Medium (DMEM) (Gibco, no. C11995500BT) supplemented with 10% fetal bovine serum (FBS) (Biological Industries, no. 04-001-1ACS) and Pen-Strep (Biological Industries, 03-031-1B) at 37°C under 5% CO_2_.

CV-A6-Chagnchun098 was isolated from patients with HFMD in 2013 in Changchun, China (GenBank accession no. KT779412) (62). CV-A6 virus was propagated in RD cells and harvested when the CPE reached 90%. Viral titers were determined in RD cells using the microplate CPE method and calculated using the Reed-Muench method (72). Sendai virus was kindly provided by Tao Wang (Tianjin University) and Junliang Chang (Changchun Institute of Biological Products Co., Ltd.) (56).

The autophagy-lysosome inhibitor bafilomycin A1 (Baf-A1) was purchased from Millipore (Billerica, MA, USA). E-64d (HY-100229) and Pepstatin A (HY-P0018) were purchased from MedChemExpress (Monmouth Junction, NJ, USA). The proteasome inhibitor, MG132, was purchased from Selleck (Houston, TX, USA).

### Plasmids

VR1012-CV-A6-2C-wt-HA (62) and VR1012-CV-A16-2C-wt-HA (strain CC024, GenBank accession number KF055238.1) (60) have been previously described. The full-length 2C-coding region of EV-A71 (strain 063, isolated from patients with HFMD in 2010) (69) was cloned into the SalI and BamHI sites of the VR1012 vector with a C-terminal HA tag. The full-length 2C-coding region of CV-B3 (strain LRY007, GenBank accession number KX981987.1) was synthesized by Generay Biotech Co., Ltd. (Shanghai, China) and cloned into the VR1012 vector between the SalI and BamHI sites. All truncated 2C_CV-A6_ mutants (1–113, 21–329, 46–329) were amplified from the CV-A6-2C-wt-HA plasmid and inserted into VR1012 between the XbaI and BamHI sites. CV-A6-2C-Δ21–25, Δ26–30, Δ31–35, Δ36–40, and Δ41–45 were constructed from CV-A6-2C-wt-HA using PCR-based site-directed mutagenesis. A similar technique was used to generate CV-A6-2C point mutations including 2C-S26A, K27A, F28A, I29A, D30A, S13N, N23S, R41K, V75A, and I96V. All mutant constructs were sequenced by Comate Bioscience Co. Ltd. (Jilin, China) to verify their correctness. Unless otherwise indicated, all 2C-wt used or illustrated in this study represents the wild-type 2C protein of CV-A6.

The C-terminal myc-tagged full-length MDA5 and RIG-I expression vectors have been previously described (73). Subsequently, full-length MDA5- and RIG-I-expressing plasmids without a tag were constructed based on the corresponding myc-tagged plasmids.

To generate a firefly luciferase-based IFNB promoter reporter plasmid (IFNB-Luc), a 2,020-bp-long fragment upstream of the IFN-β-coding region was amplified using cDNA from Jurkat cells and inserted into the pGL3-Basic vector (Promega, Madison, WI, USA) via the XhoI and HindIII sites. The IFN-β open reading frame was obtained from total RNA extracted from HEK293T cells and inserted into the SalI and XbaI sites of the VR1012 vector with a C-terminal myc tag.

All primers are shown in Table 1.

**TABLE 1:** Primers used in this study^*a*^

Primer	Sequence (5′−3′)
EV-A71-2C-HA-F (SalI)	ACGC ***GTCGAC*** ACCATGAGCGCTTCCTGGCTCA
EV-A71-2C-HA-R (BamHI)	CGC ***GGATCC*** TCACGCGTAATCTGGGACGTCGTAAGGGTATTG GAAAAGAGCCTCGATT
CV-A6-2C-wt-HA-F (XbaI)	CTAG ***TCTAGA*** ATGAGCGCCTCTTGGCTTAAG
CV-A6-2C-21–329-HA-F (XbaI)	CTAG ***TCTAGA*** ACCATGATTTCTAACAAGATTAGTAAATTCATTG
CV-A6-2C-46–329-HA-F (XbaI)	CTAG ***TCTAGA*** ACCATGTTCTTAAACAACTTGAAACAGCTAC
CV-A6-2C-wt-HA-R (BamHI)	CGC ***GGATCC*** TCACGCGTAATCTGGGACGTCGTAAGGGTA CTGAAACAAAGCTTCAATTGTATT
CV-A6-2C-1–113-HA-R (BamHI)	CGC ***GGATCC*** TCACGCGTAATCTGGGACGTCGTAAGGGTA CTGCATGTAATTGTTCATTCTCT
CV-A6-2C-1–250-HA-R(BamHI)	CGC ***GGATCC*** TCACGCGTAATCTGGGACGTCGTAAGGGTA CACTTCAATGTCACAATCCATAT
CV-A6-2C-1–311-HA-R (BamHI)	CGC ***GGATCC*** TCACGCGTAATCTGGGACGTCGTAAGGGTA TATGAGTTCAGAGATCACTGTATCC
CV-A6-2C-Δ−21–25-F	AGGGATTGGAATGGAGTAAATTCATTGACTGG
CV-A6-2C-Δ−21–25-R	CCAGTCAATGAATTTACTCCATTCCAATCCCT
CV-A6-2C-Δ−26–30-F	TGGATTTCTAACAAGATTTGGCTCAAAGAGAAGA
CV-A6-2C-Δ−26–30-R	TCTTCTCTTTGAGCCAAATCTTGTTAGAAATCCA
CV-A6-2C-Δ−31–35-F	AGATTAGTAAATTCATTGACATCATACCAGCAGCC
CV-A6-2C-Δ−31–35-R	GGCTGCTGGTATGATGTCAATGAATTTACTAATCT
CV-A6-2C-Δ−36–40-F	CTGGCTCAAAGAGAAGAGGGAGAAGGTCGAG
CV-A6-2C-Δ−36–40-R	CTCGACCTTCTCCCTCTTCTCTTTGAGCCAG
CV-A6-2C-Δ−41–45-F	GATCATACCAGCAGCCTTCTTAAACAACTTGAAAC
CV-A6-2C-Δ−41–45-R	GTTTCAAGTTGTTTAAGAAGGCTGCTGGTATGATC
CV-A6-2C-S26A-F	GATTTCTAACAAGATTGCAAAATTCATTGACTG
CV-A6-2C-S26A-R	CAGTCAATGAATTTTGCAATCTTGTTAGAAATC
CV-A6-2C-K27A-F	TTCTAACAAGATTAGTGCATTCATTGACTGGCTC
CV-A6-2C-K27A-R	GAGCCAGTCAATGAATGCACTAATCTTGTTAGAA
CV-A6-2C-F28A-F	TCTAACAAGATTAGTAAAGCAATTGACTGGCTCAAAG
CV-A6-2C-F28A-R	CTTTGAGCCAGTCAATTGCTTTACTAATCTTGTTAGA
CV-A6-2C-I29A-F	AAGATTAGTAAATTCGCAGACTGGCTCAAAGAG
CV-A6-2C-I29A-R	CTCTTTGAGCCAGTCTGCGAATTTACTAATCTT
CV-A6-2C-D30A-F	GATTAGTAAATTCATTGCATGGCTCAAAGAGAAG
CV-A6-2C-D30A-R	CTTCTCTTTGAGCCATGCAATGAATTTACTAATC
CV-A6-2C-S13N-F	AATTCAATGATATGGCCAATGCTGCCAAGGGAT
CV-A6-2C-S13N-R	ATCCCTTGGCAGCATTGGCCATATCATTGAATT
CV-A6-2C-N23S-F	GATTGGAATGGATTTCTAGCAAGATTAGTAAATTCATT
CV-A6-2C-N23S-R	AATGAATTTACTAATCTTGCTAGAAATCCATTCCAATC
CV-A6-2C-R41K-F	ATCATACCAGCAGCCAAGGAGAAGGTCGAGTTC
CV-A6-2C-R41K-R	GAACTCGACCTTCTCCTTGGCTGCTGGTATGAT
CV-A6-2C-V75A-F	ACAAGAAGATCTTGAAGCCATGTTTGGGAATGTG
CV-A6-2C-V75A-R	CACATTCCCAAACATGGCTTCAAGATCTTCTTGT
CV-A6-2C-I96V-F	CCAGCCACTGTACGCTGTAGAAGCTAAAAGAGTTTACGC
CV-A6-2C-I96V-R	GCGTAAACTCTTTTAGCTTCTACAGCGTACAGTGGCTGG
CV-A6-2C-V303I-F	CAAGGTCAGATACAGCATTGATACAGTGATCTCTGAAC
CV-A6-2C-V303I-R	GTTCAGAGATCACTGTATCAATGCTGTATCTGACCTTG
MDA5-no-tag-F (SalI)	GC ***GTCGAC*** ACCATGTCGAATGGGTATTCCACA
MDA5-no-tag-R (BamHI)	CGC ***GGATCC*** CTAATCCTCATCACTAAATAAACAG
RIG-I-no-tag-F (SalI)	ATGGGTCTTTTCTGCAGTCACCGTC ***GTCGAC*** ACCATG ACCACCGAGCAGCG
RIG-I-no-tag-R (BamHI)	AACTAGAAGGCACAGCAGATCT ***GGATCC*** TCATTTGG ACATTTCTGCTGGATCAAATGGT
IFNB-Luc-F (XhoI)	CCG ***CTCGAG*** ATGCTCATAATAGAACATTTTAAAT
IFNB-Luc-R (HindIII)	CCC ***AAGCTT*** GTTGACAACACGAACAGTGTC
pBSK-CV-A6-wt-1F (XhoI)	GGTACCGGGCCCCCC ***CTCGAG*** TAATACGACTCACTATAG TTAAAACAGCCTGTGGGTTGTAC
pBSK-CV-A6-wt-1R	TTGGTACTCTGCTGTTTGTTGGA
pBSK-CV-A6-wt-2F	AACAAACAGCAGAGTACCAAAATGA
pBSK-CV-A6-wt-2R	CTGAAACAAAGCTTCAATTGTATTGC
pBSK-CV-A6-wt-3F	CAATTGAAGCTTTGTTTCAGGGC
pBSK-CV-A6-wt-3R (BamHI)	CGCTCTAGAACTAGT ***GGATCC*** TTTTTTTTTTTTTT TTTTTTTTTTTTTTTTGCTATTCTGGTTATAACAAATTTACCCC
IFN-β-myc-F (SalI)	ACGC ***GTCGAC*** ACCATGACCAACAAGTGTCTCCTCC
IFN-β-myc-R (XbaI)	GC ***TCTAGA*** TCAAAGATCTTCTTCTGATATGAGTTTT TGTTCGTTTCGGAGGTAACCTGTAAGTCT
ACTB-RT-F	ACCGAGCGCGGCTACAG
ACTB-RT-R	CTTAATGTCACGCACGATTTCC
IFNB-RT-F	CACGACAGCTCTTTCCATGA
IFNB-RT-R	AGCCAGTGCTCGATGAATCT
CV-A6-RT-F	GGAACTGGTTTTACTGATGCAGTG
CV-A6-RT-R	TACAGGTTCAATACGGTGTTTGC

^*a*^ The restriction enzyme sites used for cloning are indicated in bold and italic. The C-terminal HA or myc tag sequence in the primers is underlined.

Transfections of 70%–80% confluent HEK293T cells or RD cells were performed using Lipofectamine 3000 (Invitrogen) according to the manufacturer’s protocol.

### Construction of infectious CV-A6 clone

To construct the plasmid of the infectious clone of the CV-A6-Changchun098 strain, RNA was extracted from the supernatant of virus-infected RD cells and then reverse transcribed to obtain cDNA. The resulting cDNA was used to amplify three viral fragments covering the full-length CV-A6-Changchun098 genome, with a T7 promoter and a poly A tail at the N- and C-termini (61). These PCR products were cloned into pBlueScript SK(+) (YouBio, VT1892, China) at the XhoI and BamHI sites using the pEASY-Basic Seamless Cloning and Assembly Kit (TransGen, China) according to the manufacturer’s instructions and designated as pBSK-CV-A6-wt. The single-point mutation infectious clone, pBSK-CV-A6-2C-F28A, was obtained by site-directed mutagenesis using 2× Phanta Max Master Mix (Vazyme, P515-01, Nanjing, China), with pBSK-CV-A6-wt as the template. All the infectious clones were confirmed by sequencing the entire genome.

To rescue viruses, HEK293T cells seeded in a T25 flask were co-transfected with linearized pBSK-CV-A6-wt (4 µg) or mutant infectious clone, which had been digested with BamHI, and pcDNA3.1-T7 DNA Pol [2 µg, kindly provided by Dr. Zhaolong Li (Jilin University, China)] using Lipofectamine 3000 reagent according to the manufacturer’s protocol (61, 74). At 6 h post-transfection, the cells were washed, and the medium was replaced with fresh medium containing 10% FBS. When approximately 90% of the cells exhibited CPE, the media and cells were harvested together, frozen at −80°C, and thawed. Freeze thaw was repeated three times to release the viruses. The samples were then centrifuged at 12,000 *g* for 30 min at 4°C to remove cellular debris, and the clear supernatant was then transferred to a new tube, aliquoted, and stored at −80°C.

### Luciferase reporter assay

The TransDetect Single-Luciferase Reporter Assay Kit (TransGen, FR101-01) was used to detect the activity of the IFNB promoter. Briefly, HEK293T cells in 24-well plates were co-transfected with 250 ng IFNB-Luc and the indicated expression plasmid or empty vector. At 24 h post-transfection, the cells were infected with SeV [20 hemagglutination (HA) units/mL] or left uninfected for 12 h. At 36 h post-transfection, the cells were washed once with PBS and lysed to measure luciferase activity, according to the manufacturer’s protocol. The readings of pGL3-transfected samples were used for background correction and are not shown.

### Enzyme-linked immunosorbent assay

IFN-β secretion in the cell culture medium was assessed using a human IFN-β enzyme-linked immunosorbent assay kit (Shanghai MLBIO Biotechnology, Shanghai, China) according to the manufacturer’s instructions.

### Co-immunoprecipitation

The co-IP experiments were performed as previously reported with minor modifications (75). HEK293T cells in six-well plates were transfected with 2C- (400 ng), MDA5-, or RIG-I-expressing vectors (1,200 ng) and then harvested at 48 h post-transfection. The cells were then washed once with cold 1× PBS, followed by disruption with lysis buffer [50 mM Tris-HCl (pH 7.5), 150 mM NaCl, and 0.5% NP-40, supplemented with complete protease inhibitor cocktail (Roche)]. The samples were sonicated at 15% power for 90 s with a 1 s break every 1 s and then centrifuged at 12,000 *g* for 30 min at 4°C to obtain a clear supernatant. These input samples were then incubated with anti-HA magnetic beads (Thermo Pierce, 88837) overnight and washed six times with cold wash buffer [20 mM Tris-HCl (pH 7.5), 100 mM NaCl, 0.1 mM EDTA, and 0.05% Tween 20]. The samples were then eluted with cold 100 mM glycine-HCl (pH 2.5) and analyzed by western blotting to detect the proteins of interest.

### Quantitative reverse transcription PCR

Total RNA was extracted from cells using the FastPure Cell/Tissue Total RNA Isolation Kit (Vazyme, RC101) and then subjected to reverse transcription with MonScript RTIII All-in-One Mix (Monad, RN05004M, Suzhou, China) according to the manufacturer’s instructions. SYBR Green-based qPCR was performed on a Cobas z480 instrument (Roche, Rotkreuz, Switzerland) using MonAmp ChemoHS qPCR Mix (Monad, RN04001N) and specific primers. Each 20 µL of reaction mixture contained 4 µL of 5 × RTIII All-in-One Mix, 0.4 µL of oligonucleotide primer (10 µM each), and 2 µL of the cDNA template. The cycling conditions were as follows: 50°C for 2 min and 95°C for 10 min, followed by 45 cycles consisting of 95°C for 30 s and 60°C for 1 min. Relative mRNA levels were normalized to the level of *ACTB* mRNA (data not shown). All the primers used for qRT-PCR are listed in Table 1.

### Western blotting and antibodies

Briefly, harvested cells were lysed in radioimmunoprecipitation assay lysis buffer [50 mM Tris-HCl (pH 7.5), 150 mM NaCl, 1% NP-40]. The lysate was supplemented with 1× loading buffer [0.08 M Tris-HCl (pH 6.8), with 2.0% SDS, 10% glycerol, 0.1 M dithiothreitol, and 0.2% bromophenol blue] and boiled at 100°C for 10 min (76). Total cell extracts were then subjected to 10% SDS-PAGE and transferred onto a polyvinylidene fluoride (PVDF) membrane (Millipore, Ireland) or nitrocellulose (NC) membrane (GE Healthcare, Germany) for western blotting analysis. After blocking with 5% nonfat dry milk, the PVDF membranes were incubated with primary antibodies, followed by incubation with the corresponding horseradish peroxidase (HRP)-conjugated secondary antibody diluted 1:10,000. Protein bands were visualized using an Immobilon Western Chemiluminescent HRP Substrate Kit (Millipore, MA, USA; catalog no. WBKLS0100) and imaged using the c500 Azure system. The NC membranes were incubated with alkaline phosphatase-conjugated anti-rabbit (Jackson, USA) or anti-mouse (Jackson, USA) secondary antibodies, and the blots were incubated with 5-bromo-4-chloro-3-indolylphosphate (Roche, Switzerland) and nitro-blue tetrazolium (Sigma-Aldrich, USA).

The following primary antibodies were used: anti-tubulin (TransGen, no. HC101-02), anti-HA.11 epitope tag (BioLegend, no. 901513), anti-myc tag, clone 4A6 (Millipore, no. 05–724), anti-MDA-5 (D74E4) rabbit mAb (Cell Signaling Technology, no. 5321), anti-RIG-I (D14G6) rabbit mAb (Cell Signaling Technology, no. 3743), and anti-CV-A6 rAb (GeneTex, no. GTX87102). HRP-conjugated goat anti-rabbit IgG (no. NEF812001EA) and goat anti-mouse IgG (no. NEF822001EA) antibodies were obtained from PerkinElmer. All antibodies were used in accordance with the manufacturer’s instructions.

### Viral infection and one-step growth curves

Cells were infected with the virus at the indicated multiplicity of infection. The unbound virus was removed 2 h after absorption, and the cells were washed twice in PBS (pH 7.4). Fresh DMEM containing 2% FBS was added, and the cells were incubated at 37°C in 5% CO_2_ for the indicated time points. The medium of each sample was used to extract RNA for qRT-PCR, and the cell lysate was used for detecting VP1 expression by western blotting.

One-step growth assays were performed as previously described (62).

### Statistical analysis

All data are presented as the mean ± SD from three independent experiments. Data were analyzed using unpaired two-tailed Student’s *t*-tests in Microsoft Excel. All plots were analyzed using GraphPad Prism 8.0.2 (GraphPad Software, Inc., San Diego, CA, USA). Significance was set at *P* < 0.05.
